# Comparative assessment of *Brassica nigra* seed and its sprout ethanolic extracts against paracetamol-induced hepatotoxicity in rats: insight into antioxidant and anti-apoptotic pathways

**DOI:** 10.3389/fnut.2026.1807243

**Published:** 2026-04-30

**Authors:** Seham S. Alqaraawi, Mona S. Almujaydil

**Affiliations:** Department of Food Science and Human Nutrition, College of Agriculture and Food, Qassim University, Buraydah, Saudi Arabia

**Keywords:** antioxidants, apoptosis, *Brassica nigra* seed, *Brassica nigra* sprout, hepatoprotection, oxidative stress, paracetamol, phytochemical

## Abstract

**Background:**

The liver’s central role in xenobiotic metabolism renders it highly susceptible to injury from oxidative stress. Therapeutic drug solutions for liver diseases remain limited. Natural medicinal plants present an option; sprouting is an emerging trend to maximize the plants’ bioactivity. This study explores, for the first time, a direct comparative assessment of the hepatoprotective effects of *Brassica nigra* (BN) seeds versus their sprout ethanolic extracts against paracetamol-induced hepatotoxicity.

**Methods:**

Wistar rats were grouped into (1) negative control and (2) positive control. Rats in groups 3, 4, and 5 were gavaged with 100 mg/kg BW silymarin (drug control), 500 mg/kg BW of *Brassica nigra* (BN) seed ethanolic extract, and 500 mg/kg body weight BN sprout ethanolic extract, respectively, for 21 days. On day 22 of the experiment, all groups except the negative group were challenged with a single high dose of 3 g/kg paracetamol (APAP) orally.

**Results:**

A high dose of APAP induced severe hepatotoxicity, evident in elevated serum liver enzymes, dyslipidemia, oxidative stress, and apoptosis. BN seed and BN sprout extracts attenuated hepatotoxicity, although the BN sprout extract demonstrated significantly superior efficacy. It more effectively normalized lipid profiles, rebalanced liver enzymes, particularly ALT and *γ*-GT, improved antioxidant enzyme activities (catalase, SOD, and glutathione peroxidase), and reduced malondialdehyde. The apoptotic indicators cytokeratin-18 and caspase-cleaved cytokeratin-18 were also markedly decreased by BN sprout extract.

**Conclusion:**

BN sprout extract exhibits significantly stronger hepatoprotective effects than its seed counterpart against APAP-induced acute liver injury. The pathway mechanism is attributed to enhanced antioxidants and anti-apoptotic activities, likely due to the higher concentration and bioavailability of phytochemicals achieved through the sprouting process. *Brassica nigra* sprout extract merits further clinical research to investigate its safety and therapeutic potential.

## 1 Introduction

The liver is a primary metabolic center that performs vital homeostatic functions. These include macronutrient metabolism and the detoxification of xenobiotics, such as those found in cigarette smoke, environmental contaminants, and drug metabolites (1, 2). Precisely because of this central role in detoxification, the liver is extremely vulnerable to oxidative stress-related harm (3). During detoxification, the generation of free radicals can cause frequent oxidative damage to liver cells, a process that may lead to serious conditions, including hepatitis and cirrhosis (4–6). Liver disease affects approximately one billion individuals worldwide and is responsible for two million deaths annually (7). The international prevalence among patients with liver disease ranges between 11 and 20% (8). Non-alcoholic liver disease impacts between 6 and 30% percent of adults around the globe (3). Paracetamol (acetaminophen or APAP) is a safe antipyretic analgesic substance when used appropriately and in accordance with recommended therapeutic dosages (9). However, both humans and animals may have acute liver failure or severe hepatic damage by APAP overdose (10, 11). The induction of hepatotoxicity by APAP in laboratory animal models is a widely used method for studying hepatic injury and evaluating antioxidant agents (12, 13). On the other hand, *Silybum marianum* (silymarin) is a natural source of flavoligands and the flavonoid taxifolin, commonly called milk thistle. Silymarin is a natural compound that provides effective protection against hepatic toxicity (14). Recently, silymarin has been recognized as a valuable hepatoprotective agent in the pharmaceutical industry and is used as a standard reference in laboratory research (15). The current study chose APAP to induce hepatotoxicity and silymarin as a drug reference to protect against liver damage.

Given that no synthetic pharmaceutical drugs effectively halt the progression of liver disease and that current therapeutic options remain limited (6), plant-based medicines have garnered renewed interest for the management of hepatic disorders (16). Natural medicinal plants produce a vast array of secondary metabolites, such as antioxidants, which can exert cytoprotective effects beneficial in preventing or treating liver damage (17). Preclinical research demonstrates that phytochemicals can mitigate liver injury in various experimental models. The Brassicaceae (Cruciferous family) includes broccoli, cabbage, cauliflower, Brussels sprouts, radish, and mustard, and has been extensively studied for its health-promoting phytochemicals (18). Cruciferous vegetables are well known for their rich nutrients and health-promoting bioactive compounds. Cruciferous plants have anti-inflammatory and anti-tumor properties (19). *Brassica nigra* (L.) Koch (khardal, Black Mustard) (BN) is an annual plant in the Brassicaceae family. *Brassica nigra* is distinct for its exceptionally high concentration of sinigrin (SIN), the glucosinolate (GSLs) precursor to allyl isothiocyanate (AITC) (20). AITC is a potent bioactive compound recognized for its antioxidant and anti-inflammatory properties (21, 22). A recent study by Rahman et al. (23) indicated that BN seeds have many phytochemicals, such as flavonoids, alkaloids, vitamins, and minerals, suggesting a yet-to-be-fully-explored therapeutic potential. BN has shown a protective role against liver toxicity caused by cadmium chloride (24).

Sprouting is a natural process that markedly alters the nutritional and phytochemical composition of seeds by breaking down complex macromolecules into simpler, more digestible forms (22, 25). In addition, sprouting enhances the concentration and bioavailability of phytochemical components such as glucosinolates, isothiocyanates, and phenolic compounds and promotes the synthesis of new bioactive constituents (5, 26), as well as other nutrients, including essential amino acids and micronutrients (4). Furthermore, Brassica sprouts are particularly rich in SIN, a precursor of AITC, more than Brassica, which has been shown to protect organs such as the liver, kidney, and intestine (27).

Despite the recognized nutritional benefits of BN seeds, the pharmacological potential of their sprouts remains comparatively unexplored. Although *Brassica nigra* seeds have demonstrated some hepatoprotective potential, the pharmacological potential of BN sprouts has never been investigated. No study has directly compared the hepatoprotective efficacy of seeds and sprouts against liver injury, leaving unanswered whether sprouting, a bioprocessing technique known to enhance bioactive compounds, confers superior protective effects. Comparative phytochemical profiling of BN seeds and sprouts has not been reported, limiting understanding of the compositional changes underlying differential bioactivity. Furthermore, the anti-apoptotic effects of BN sprouts have not been explored. Emerging evidence suggests BN may not be entirely innocuous due to the presence of anti-nutritional factors and limited safety data (28). To address these gaps, this study is designed to conduct, for the first time, a direct comparative assessment of the hepatoprotective effects of BN seed versus sprout ethanolic extracts in a rat model of paracetamol-induced acute hepatotoxicity. Furthermore, this study provides a unique, comprehensive comparative analysis of the phytochemical profiles of both extracts, elucidating their underlying mechanisms, with a focus on antioxidant and anti-apoptotic pathways.

## 2 Materials and methods

### 2.1 Ethical standard

The Committee of Health Research Ethics approved the current work, Qassim University, KSA (approval number 24-06-04), in compliance with International Animal Ethics, and in accordance with the ARRIVE Essential 10 criteria.

### 2.2 *Brassica nigra* seeds

Organic seeds of BN were obtained from Baharat Dukkani Company, Turkey, which holds ISO 9001:2015 and ISO 22000:2005 certifications. The organic seeds (Voucher Specimen ID: ANK123456) were stored in a dry, cold environment without the use of preservatives and in firmly sealed bags. The seeds were separated into three parts: one part was used for the ethanolic extraction of BN seeds, the second for sprouting, and the third for the subsequent ethanolic extraction of BN sprouts. The third part of BN seeds, together with BN sprouts, was stored separately in dark packages to determine total phenols, total flavonoids, antioxidants, and phytochemical constituents.

### 2.3 *Brassica nigra* sprouting

The sprouting process of BN occurs in three stages: soaking, sprouting, and drying. First, dry BN seeds were sterilized in a 0.7% sodium hypochlorite solution (v/v) for 5 min at room temperature. The seeds were rinsed several times under running distilled water and soaked for 12 h in cool water at room temperature. Sprouting was done using the traditional method, “The towel and baggy method for germinating seeds” (29). In summary, seeds were washed with sterile distilled water, spread between two layers of wet towels, and covered. BN seeds were irrigated twice daily. The temperature and relative humidity were maintained at 22 ± 2 °C and 95%, respectively (30), under indirect light with good ventilation (31). On the fifth day, sprouts were harvested to obtain the maximum total phenol and flavonoid contents and the highest oxidative activities (22). They were thoroughly rinsed and drained (30). The buds of the sprouted seeds measured approximately 5–6 cm. A hot air oven (Model ACT1410, India) was used to dry sprouted seeds at 40 °C for 24 h, ground in an electric grain mill (Guangzhou Foodsense, Catering Equipment Co., Ltd., China), and sieved through a 0.6 mm sieve. Sprout powder was divided into two portions: one stored in dark packages for phenolic, flavonoid, antioxidant, and phytochemical analyses, and the other used for ethanolic extraction for oral supplementation.

### 2.4 *Brassica nigra* (seed and sprout) ethanolic extraction

Following the method of Cho and Kim (32) with modifications, ground BN seeds and BN sprout powder were extracted separately using a hydroethanolic solution (70% ethanol, 30% water) at a 1:5 (w/v) ratio (Arkan, Germany). Samples were then shaken for approximately 3 days in a shaking water bath at room temperature. The solutions were filtered through a muslin cloth. The filtrates were subsequently centrifuged at 5000 rpm for 30 min. After filtration through Whatman No. 1 filter paper, the supernatants were concentrated using a rotary evaporator (KNF Technology Shanghai Co., Ltd., China) to obtain the BN seed and sprout extracts. Extracts were suspended in distilled water to a concentration of 100 mg/mL, transferred to glass vials, and stored at 4 °C.

### 2.5 Total phenols and total flavonoids of *Brassica nigra* (seeds and sprouts)

The adjusted temperature was 20 °C with 37% relative humidity, maintained as the regulated environmental condition for the use of a UV/Vis spectrophotometer (Jenway, England). Calorimetrically, the Folin–Ciocalteu reagent was used to measure total phenols. Gallic acid equivalents per kilogram of sample were used to calculate the total phenolic content using the formula for regression from the typical curve (33) (*y* = 1,001.1x + 4.4832, r2 = 0.9993). The total flavonoid content was estimated using the aluminum chloride colorimetric technique (34). One milliliter of the tested sample was mixed with 5.6 milliliters of distilled water, 0.2 milliliters of aluminum chloride (10%), 1 M potassium acetate (0.2 milliliters), and 3 milliliters of methanol. The combination was then left to rest for 30 min at room temperature. Absorbance was measured at 420 nm. A total of 1 mg/mL of Rutin standards was used for calibration. The total flavonoid content (*y* = 365.26x − 6.1589, r2 = 0.9975) was calculated using the regression equation from the standard curve and reported as rutin equivalents in milligrams per 100 grams of sample.

### 2.6 Antioxidant activity of *Brassica nigra* (seeds and sprouts)

The 2,2-diphenyl-1-picrylhydrazyl (DPPH) radical scavenging activity assay was performed according to the method of Brand-Williams et al. (35). The BN seed and sprout extracts were dissolved in methanol to prepare four concentrations: 0.2, 0.5, 1.0, and 2.0% (w/v). Briefly, 100 μL of each sample concentration was mixed with 100 μL of a 0.2 mM DPPH radical solution prepared in methanol. The mixture was thoroughly stirred and incubated in the dark for 15 min at room temperature. Absorbance was measured at 517 nm against a methanol blank. The test was run at 25 °C and 38% relative humidity. Using the formula [(Ao – A1)/Ao] × 100, since A1 considers the absorbance with the sample and Ao considers the absorbance without the sample, the percentage of scavenging effect was determined.

### 2.7 Bioactive phytochemical constituents profiling of BN seeds and BN sprouts

High-performance liquid chromatography (HPLC) was used to quantify individual phenolic and flavonoid compounds in the ethanolic extracts of BN seeds and BN sprouts. The analysis was performed using an Agilent 1260 Infinity HPLC Series (Agilent, USA) equipped with a quaternary pump and a Kinetex^®^ (Phenomenex, USA) 1.7 μm EVO C18 column (50 mm × 2.1 mm). The column temperature utilized was 30 °C. A ternary linear elution gradient was created using (1) Grade water, HPLC with 0.1% H3PO4 (v/v), (2) acetonitrile with 0.1% H3PO4 (v/v), and (3) 0.2 mL/min, a methanol flow rate. Compounds were identified by comparing retention times with authentic standards (Sigma-Aldrich, USA). Quantification was performed using calibration curves prepared from standard solutions of each compound. Analyses were performed in triplicate for three independent plant samples. The following compounds were targeted: gallic acid, p-hydroxybenzoic acid, catechin, vanillic acid, caffeic acid, chlorogenic acid, syringic acid, p-coumaric acid, ferulic acid, o-coumaric acid, rutin, hesperidin, resveratrol, myricetin, rosmarinic acid, quercetin, kaempferol, and apigenin.

### 2.8 Silymarin (*Silybum marianum*)

Silymarin was purchased from NOW FOODS (Bloomingdale, IL, USA). The product was standardized to contain 280 mg of silymarin flavonoids per tablet, with a purity of 80% (i.e., 80% of the extract consists of silymarin flavonoids). Silymarin was chosen to use in the present study as a standard reference drug. This natural compound is a well-known hepatoprotective agent and was administered at a dose of 100 mg/kg body weight.

### 2.9 Acute hepatotoxicity study

A pilot experiment was conducted to determine the optimal dose of acetaminophen (APAP) for inducing hepatotoxicity in rats. Rats were randomly allocated into four groups (*n* = 3 per group): a control group receiving a vehicle. The other three treatment groups received a single oral dose of APAP at 1, 2, or 3 g/kg, respectively, dissolved in a 40% sucrose solution. Forty-eight hours after APAP administration (36), blood samples were taken, and liver enzymes, alanine aminotransferase (ALT), and aspartate aminotransferase (AST) were measured to evaluate liver function. Animals were euthanized under ether anesthesia, and their livers were quickly collected for histological analysis. Administration of APAP at 3 g/kg BW produced the most pronounced liver injury, characterized by marked necrosis and hepatocellular degeneration, as well as the peak elevations in ALT and AST, indicative of severe hepatotoxicity. Consequently, APAP at 3 g/kg was recommended for inducing liver injury in this study. Fortunately, this dose has been shown in several studies (37) to induce hepatotoxicity effectively.

### 2.10 Induction of hepatotoxicity

Paracetamol, also known as N-acetyl-para-aminophenol (APAP), marketed under the name Panadol, was used in this study to induce acute hepatic injury. The APAP was sourced as a 500 mg paracetamol tablet, designed with OPTIZORB Technology (Haleon Ireland Limited, Clocherane, Youghal Road, Dungarvan, Co. No. 15513, Waterford, X35 Y983, PM-IE-PAN-22-00137, Ireland). To induce acute hepatotoxicity, animals received a single high oral dose of APAP, resulting in an acute toxicity test; 3 g/kg dissolved in 40% sucrose buffer on day 22 of the experiment. A similar APAP dose was also used in several studies (37).

### 2.11 Experimental animal care

Healthy 8-week-old adult Wistar rats with an average weight of 200 ± 15 g were acquired from the University of King Saud laboratory center, Riyadh, KSA, which is part of the Animal Husbandry Department. The animals were relocated to an adjustable rearing room at the Department of Food Science and Human Nutrition, College of Agriculture and Food, Qassim University, KSA. The rats were kept in wire cages for a week to acclimate to their new surroundings before the experiment began, with five rats per cage. For optimal efficiency, the rearing circumstances included a 12-h light/dark cycle, 50% ± 5% relative humidity, and a temperature of 22–23 °C. The animals were provided with fresh water and a simple commercial diet based on their needs during the trial. The purchased diet was supplied by “Wafi” commercial animal feed company (Qassim, KSA) and was formulated according to the nutritional standards established by the National Research Council (38). This study was conducted following the animal care guidelines and ethics established by the Deanship of Scientific Research at Qassim University and reported in accordance with the 10 ARRIVE guidelines.

### 2.12 Experimental protocol and sampling

A total of 50 male Wistar rats were randomly divided into five experimental groups, with 10 rats per group (*n* = 10 per group). This sample size was chosen based on previous studies that investigated the hepatoprotective effects of plant extracts in rodent models of paracetamol-induced hepatotoxicity (15, 37, 39). This group size ensured adequate statistical power to detect meaningful differences across the measured parameters while adhering to the 3Rs principles of ethical animal research. This sample size achieved differences between groups and confirmed it was adequate to identify meaningful biological effects while controlling Type II error. The statistical analysis was used to control the family-wise Type I error rate, which minimizes the risk of false-positive findings despite the large number of parameters assessed. Group 1 served as the negative control, and Group 2 as the positive (model) control; both groups received no supplementation for 21 days. Group 3 received silymarin via oral gavage at a dose of 100 mg/kg body weight, which served as the drug control. This dose was calculated as a human-equivalent dose using a standard conversion equation (40) and falls within the typical 15–20 mg/kg human dosage range, consistent with recommendations from several studies (15, 41). Group 4 administered 500 mg/kg BW of BN seed ethanolic extract (42, 43). *For a fair comparison*, Group 5 received the same dose (500 mg/kg BW) of BN sprout ethanolic extract for 21 days. On day 22 of the experiment, all groups except the negative control group were challenged orally with a single overdose of APAP (3 g/kg BW), selected based on preliminary acute hepatotoxicity results, and according to the method of Chariyakornkul et al. (37), dissolved in 40% sucrose buffer. The first negative group was gavaged only with a 40% sucrose buffer vehicle.

Blood was drawn via the ocular route (performed by a trained technician under light diethyl ether anesthesia at a concentration of 5–10% via inhalation in a controlled chamber for 2–3 min until adequate sedation was achieved, as confirmed by loss of pedal reflex) 48 h after APAP administration. Serum was collected using centrifugation at 2,000 × g for 10 min. The collected serum was stored at −20 °C for subsequent biochemical analyses. For biochemical analyses, blood samples were collected from all 10 rats in each group (*n* = 10 per group), including serum parameters, lipid profile, and liver enzyme assays. Meanwhile, the sample size of serum used is 5 for apoptosis markers. From each group, three animals were selected randomly, anesthetized using diethyl ether (5–10% inhalation, as described above), and then euthanized by cervical dislocation (a physical method to ensure rapid cessation of vital functions). Every effort was made to minimize animal suffering throughout the study. All procedures were performed under light anesthesia to reduce stress and discomfort, and animals were handled gently by trained personnel. Death was confirmed by the absence of heartbeat, respiration, and corneal reflex for at least 2 min. The liver was carefully excised, placed on filter paper, and rinsed with ice-cold 1.15% KCl. The relative liver weight of the 3 samples was calculated as a ratio to body weight. The liver was divided into two sections: one for lipid peroxidation and antioxidant activity assays, and the other for histological examination. Throughout the study, rats were weighed weekly to determine the precise dosage of each supplement.

### 2.13 Relative liver weight

After cleaning with saline solution, the liver was weighed on an analytical balance to determine its absolute weight. The relative liver weight, expressed as a percentage of body weight, was calculated using the following formula (39):


$$\frac{\text{absolute liver weight}\;(g)}{\text{Body weight}\;(g)}\times 100$$

### 2.14 Liver homogenization

The manufacturer’s instructions were followed for preparing the liver specimens for homogenization. To summarize, liver tissue was homogenized in phosphate-buffered saline (pH 7.4) and centrifuged for 20 min at 4 °C at 1,200 × g. For analysis, the resultant supernatant was gathered (44). The Lowry technique was used to determine the protein concentration of the supernatant (45). The supernatant collected from the liver was kept for assay of antioxidant activities and lipid peroxidation.

### 2.15 Lipid profile

Serum triglyceride (TG), total cholesterol (TC), and high-density lipoprotein (HDL-C) levels were measured calorimetrically using commercial laboratory kits, CAT. NO. TR 20 30, CH 12 20, and CH 12 30, respectively. The commercial kits were acquired from Cairo, Egypt’s Biodiagnostic, Diagnostic, and Research Reagents Company. The following equations were used to calculate low-density lipoproteins (LDL-C), total lipids (TLs), and very-low-density lipoproteins (VLDL) (46, 47):

*LDL-C (mg dL/kg) =* $\text{Total cholesterol}-\mathrm{HDL}-\frac{\text{Triglycerides}\;}{5}$

*TLs (mg dL/kg) = [2.27 x Total cholesterol) + TG + 62.3]*

*VLDL conc. (mg dL/kg) =* $\frac{\text{Triglycerides}\;}{5}$

### 2.16 Liver function performance

Commercial colorimetric kits were used for the analysis of serum parameters. Liver enzymes: alanine aminotransferase (ALT; Cat. No. AL 10 32) and aspartate aminotransferase (AST; Cat. No. AS 10 62), were assayed using kits from Biodiagnostic and Research Reagents Co. (Cairo, Egypt). Meanwhile, *γ*-Glutamyltransferase (*γ*-GT) was measured using a kit from Elabscience, USA (Cat. No. E-BC-K126-M). Serum protein parameters were determined as follows: total protein (TP; Cat. No. TP 20 20) and albumin (Alb; Cat. No. AB 10 10) were analyzed using kits from Biodiagnostic and Research Reagents Co. (Cairo, Egypt). Globulin (Glob) concentration was calculated mathematically (Glob = TP – Alb), and the albumin-to-globulin ratio (A/G) was derived. Total bilirubin (T-BIL; Cat. No. BR 1111) and direct bilirubin (D-BIL; Cat. No. BR 11 12) were quantified using kits from Biodiagnostic Research Reagents Co. Blood urea nitrogen (BUN) was determined with a kit from Elabscience, USA (Cat. No. E-BC-K183-S).

### 2.17 Hepatic tissue antioxidant activities and lipid peroxidation contents

Antioxidant enzyme activities and lipid peroxidation were assessed in liver tissue using commercial colorimetric kits. Superoxide dismutase (SOD; Cat. No. SD 25 23) and glutathione peroxidase (GPx; Cat. No. GP 25 26) were measured using kits from Biodiagnostic and Research Reagents Co. (Cairo, Egypt). Catalase (CAT) activity was determined with a kit from Elabscience, USA (Cat. No. M-BC-K031-S). Lipid peroxidation was evaluated by colorimetrically quantifying malondialdehyde (MDA) using a kit from Biodiagnostic and Research Reagents Co. (Cat. No. MD 25 31).

### 2.18 Apoptosis markers

Cytokeratin-18 (CK18) levels were quantified using a commercially available ELISA kit (Sunlongbiotetech, China, Cat. No. SL1216Ra). The assay sensitivity was 12 pg./mL, with intra-assay and inter-assay coefficients of variation (CV) of 0.4 and 12.6%, respectively. The inter-assay CV of 12.6% falls within the generally accepted range for ELISA-based assays (<15%), confirming acceptable reproducibility and reliability of the measurements.

Caspase-cleaved cytokeratin-18 (CK-18/M30, CCC-18) was measured using a separate ELISA kit (FineTest, Wuhan Fine Biotech Co., Ltd., China, Cat. No. EM2199). This assay had a detection range of 46.875–3,000 pg./mL; the intra-assay and inter-assay CVs were <5.07 and <4.97%, respectively.

### 2.19 Histopathological observation

The liver specimens, which had been previously stored for histological analysis, were preserved in 10% formal saline and embedded in paraffin wax. The liver sections were examined for architectural changes. The liver specimens were stained with Hematoxylin and Eosin (H&E) (48).

### 2.20 Statistical analysis

Data are presented as the mean ± standard error (SE). A one-way analysis of variance (ANOVA) was performed for each measured parameter using SAS software, version 20 (SAS Institute, USA). Where ANOVA indicated a significant effect, *post-hoc* pairwise comparisons were conducted using Tukey’s Honest Significant Difference (HSD) test. The treatment groups (BN seed and BN sprout extracts) and the negative control group were compared with the positive control (APAP-treated model). Statistical significance was set at *p* < 0.05 and *p* < 0.01.

The following linear model was applied: $Y_{\mathrm{ij}}=\mu +T_{i}+\in_{\mathrm{ij}}$ , where $Y_{\mathrm{ij}}\;$ is the observed value, μ is the overall mean, $T_{i}\;$ is the fixed effect of the i , and $\in_{\mathrm{ij}}\;$ is the random error.

## 3 Results

### 3.1 *Brassica nigra* (seeds and sprouts): contents of total phenols, flavonoids, and antioxidant activity

The total phenolic content was found to be higher in sprouts (728.38 ± 24.6 mg GAE/100 g) compared to seeds (709.95 ± 47.2 mg GAE/100 g) (Table 1). Conversely, the total flavonoid content, expressed as mg rutin equivalent per 100 g (mg RE/100 g), was higher in the seeds (792.55 ± 83.3 mg RE/100 g) than in the sprouts (732.90 ± 22.5 mg RE/100 g). Notably, none of these differences could reach statistical significance. Antioxidant activity of BN seeds and BN sprouts, as measured by the DPPH radical-scavenging assay, increased with concentration for both samples (0.20, 0.50, and 1.00%). Observantly, the BN sprouts exhibited a higher percentage of inhibition compared to the seeds. At the highest concentration (1.00%), both samples demonstrated potent antioxidant activity, with sprouts (95.33 ± 10.3%) showing higher activity than seeds (92.91 ± 15.8%).

**Table 1:** *Brassica nigra* (seeds and sprouts): contents of total phenols, flavonoids, and antioxidant activity.

*Brassica nigra*	Total phenols (mg gallic acid equivalent/100 g)	Total flavonoids (mg rutin equivalent/100 g)	Scavenging activity (DPPH%)		
			0.20%	0.50%	1.00%
Seeds	709.95 ± 47.2	792.55 ± 83.3	52.84 ± 8.3	87.59 ± 11.7	92.91 ± 15.8
Sprouts	728.38 ± 24.6	732.90 ± 22.5	66.31 ± 6.8	90.78 ± 9.6	95.33 ± 10.3

DPPH: 2,2-Diphenyl-1-picrylhydrazyl is expressed as a percentage inhibition. Mean± standard error (SE), (*n* = 3).

### 3.2 HPLC profiling of bioactive phytochemical constituents in BN seeds and BN sprouts

To provide a more characterized and detailed description of phytochemical profiling, HPLC analysis was performed to quantify individual phenolic and flavonoid compounds. The analysis showed a diverse range of phenolic and flavonoid compounds in both BN seeds and BN sprouts, with notable quantitative differences between the two forms. Detailed quantification of 17 individual compounds is presented in Tables 2, 3. The most abundant compounds in BN seeds were flavonoids such as rutin (206.05 mg/kg) and catechin (173.27 mg/kg), as well as the phenolic acid ferulic acid (235.14 mg/kg). Gallic acid was notably absent from the seed extract.

**Table 2:** HPLC profiling of bioactive phytochemical constituents in *Brassica nigra* seeds.

Phytochemicals	Expected retention time	Retention time (min)	Area	Amount (mg/kg) °
p-Hydroxybenzoic acid	4.800	4.88	67.7407	17.254
Catechin	5.700	5.61	356.7185	173.271
Vanillic acid	6.200	6.22	51.1656	8.495
Caffeic acid	6.400	6.62	25.7613	2.998
Chlorogenic acid	6.725	6.82	42.0666	13.143
Syringic acid	6.941	7.12	47.1217	4.489
p-Coumaric acid	7.400	6.93	40.7788	3.020
Ferulic	9.270	9.34	620.8693	235.144
o-Coumaric acid	9.550	9.58	106.5595	6.272
Rutin	10.200	11.24	1431.0138	206.051
Hesperidin	12.000	12.21	23.6563	4.778
Resveratrol	12.300	12.31	20.1482	5.847
Myricetin	12.600	12.69	22.2026	2.650
Rosmarinic acid	13.700	13.63	71.7203	30.463
Quercetin	14.405	14.72	4.5805	4.881
Kaempferol	16.060	16.20	9.8064	1.371
Apigenin	16.280	16.32	15.3801	0.231

To make reading easier, the standard error of the mean, which did not surpass 10% of the mean value, was eliminated from the mean of three separate plant samples taken from the exact location.

**Table 3:** HPLC profiling of bioactive phytochemical constituents in *Brassica nigra* sprouts.

Phytochemicals	Expected retention time	Retention time (min)	Area	Amount (mg/kg) °
Gallic acid	2.800	2.85	14.0193	1.401
p-Hydroxybenzoic acid	4.800	4.88	47.0193	11.976
Catechin	5.700	5.60	484.8800	235.523
Vanillic acid	6.200	6.20	67.3998	11.191
Caffeic acid	6.400	6.34	56.2955	6.551
Chlorogenic acid	6.725	6.70	75.8263	23.690
Syringic acid	6.941	6.79	54.7776	5.218
p-Coumaric acid	7.900	6.91	48.1714	3.567
Ferulic	9.270	9.31	675.5967	255.871
o-Coumaric acid	9.550	9.55	117.5804	6.921
Rutin	10.900	11.16	1677.4149	241.530
Hesperidin	12.000	11.98	69.5757	14.052
Resveratrol	12.300	12.38	108.0284	31.350
Myricetin	12.600	12.62	46.8755	5.595
Rosmarinic acid	13.700	13.77	7.4376	3.159
Quercetin	14.405	14.51	141.5450	150.823
Kaempferol	16.060	16.17	35.6934	4.989
Apigenin	16.280	16.33	21.2404	0.318

To make reading easier, the standard error of the mean, which did not surpass 10% of the mean value, was eliminated from the mean of three separate plant samples taken from the exact location.

BN sprouts exhibited substantial changes in the phytochemical profile. The most abundant compounds in BN sprouts were rutin (241.53 mg/kg), catechin (235.52 mg/kg), ferulic acid (255.87 mg/kg), and quercetin (150.82 mg/kg), representing increases of 17.2, 36.0, 8.8%, and 3,194% compared to BN seeds, respectively. Notably, quercetin concentration increased more than 30-fold, from 4.58 mg/kg in seeds to 150.82 mg/kg in sprouts. Gallic acid was detected exclusively in sprouts. Overall, the sprouting process enhanced the concentration of the quantified bioactive compounds, with a marked increase observed for several key flavonoids and phenolic acids.

### 3.3 Effect of BN seed and BN sprout extracts on body gain, absolute and relative liver weight

None of the treatments significantly affected body weight gain, indicating no adverse metabolic effects (Table 4). The positive control group, which received a high dose of APAP, showed a notable elevation of wet liver weight (absolute liver weight) (8.1 ± 0.7 g vs. 5.6 ± 0.4 g, *p* < 0.05) and relative liver weight (3.94 ± 0.25 vs. 2.46 ± 0.22, *p* < 0.05). A significant decrease (*p* < 0.05) in relative liver weight was observed in the groups receiving silymarin (the standard reference), BN seed extract, and BN sprout extract compared to the positive control group. This finding demonstrates the hepatoprotective effects of these treatments. BN sprout extract exhibited the lowest absolute liver weight (5.2 ± 0.6 g) and relative liver weight (2.32 ± 0.37%). However, a direct statistical comparison between the two extract groups revealed no significant differences in absolute or relative liver weight. These findings indicate that while both BN seed and sprout extracts effectively reduced APAP-induced liver enlargement, the observed differences between the two extracts were not statistically significant for this parameter.

**Table 4:** Effect of BN seed and BN sprout extracts on body gain, absolute and relative liver weight.

Group	Initial body wt. (g)	Final body wt (g)	Body gain (g)	absolute liver wt. (g)	Relative liver wt
Negative control	202.6 ± 8.4	224.7 ± 11.6	22.3 ± 6.1	5.6 ± 0.4	2.46 ± 0.22
Positive control + APAP	189.8 ± 5.2	208.3 ± 12.9	18.8 ± 4.5	8.1 ± 0.7*	3.94 ± 0.25*
Silymarin + APAP	207.3 ± 6.8	227.5 ± 13.5	20.9 ± 6.4	5.4 ± 1.3	2.35 ± 0.38^a^
BN seed extract + APAP	205.4 ± 7.5	231.2 ± 14.3	23.1 ± 5.8	5.5 ± 1.1	2.45 ± 0.41^a^
BN sprout extract + APAP	210.2 ± 5.5	230.4 ± 10.4	20.7 ± 5.3	5.2 ± 0.6^a^	2.32 ± 0.37^a^
*p*-value	0.1278	0.1386	0.1427	0.0429	0.0374

Values represent the mean ± standard error (SE), (*n* = 3 per group). Within a column, values marked with a signal * differ significantly (*p* < 0.05) from the negative control, while values marked with a superscript ^a^ differ significantly (*p* < 0.05) from the APAP-positive control. APAP, paracetamol; BN, *Brassica nigra*.

### 3.4 Effect of BN seed and BN sprout extracts on lipid profile

**Figure 1:**
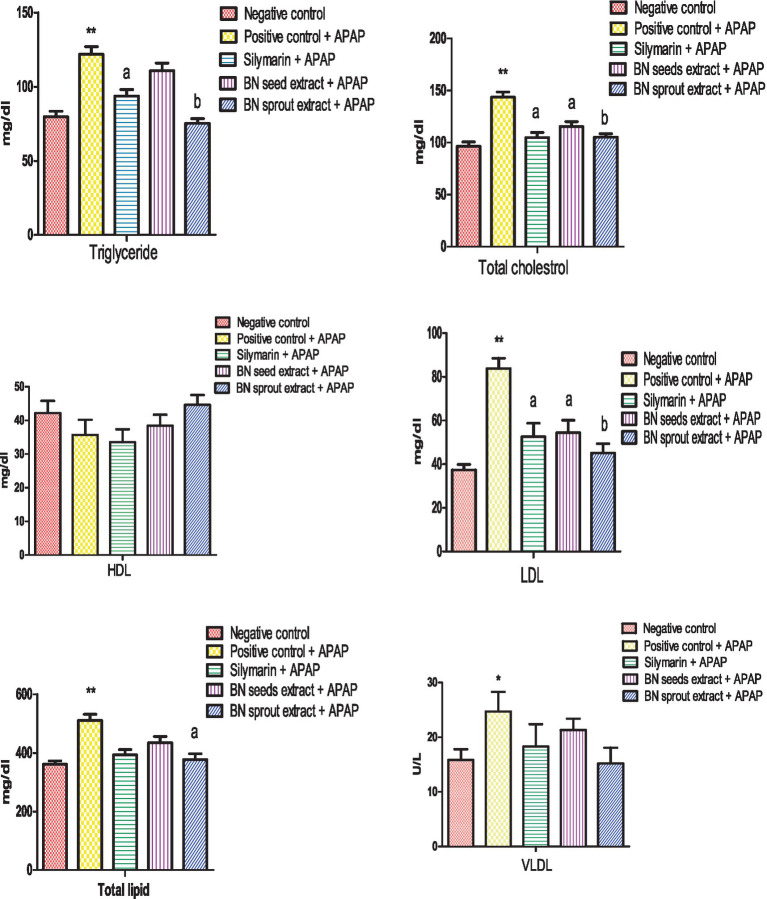
Effect of BN seed and BN sprout extracts on lipid profile. The bars point out the means ± SE (*n* = 10 per group). The signals (* and **) indicate significant differences at (*p* < 0.05 and *p* < 0.01, respectively) compared to the negative control group. The letters (a and b) indicate significant differences at *p* < 0.05 and *p* < 0.01, respectively, compared to the APAP-positive control group (one-way ANOVA followed by Tukey’s HSD *post-hoc* test). APAP, paracetamol; BN, *Brassica nigra*; TG, triglycerides; TC, total cholesterol; HDL-C, high-density lipoprotein; LDL-C, low-density lipoprotein; TL, total lipids; VLDL, very low-density lipoprotein.

Compared to the negative control, the APAP-treated positive control group showed significant disturbances in its lipid profile. Specifically, there were marked elevations (*p* < 0.01) in triglycerides, total cholesterol, LDL, and total lipids (121.8 ± 5.2, 143.6 ± 4.9 2, 83.8 ± 4.7, and 510.3 ± 21.1) mg/dL, respectively, accompanied by a significant elevation (*p* < 0.05) in VLDL (24.7 ± 2.3 U/L). Silymarin significantly reduced (*p* < 0.05) triglycerides, total cholesterol, and LDL (93.6 ± 4.4, 104.7 ± 5.1, and 52.5 ± 6.3) mg/dL compared to the positive control (Figure 1). Administering 500 mg/kg of BN seed extract significantly (*p* < 0.05) lowered serum levels of total cholesterol and LDL (115.3 ± 4.7 and 54.4 ± 5.7 mg/dL, respectively) with insignificant effect on other parameters. BN sprout extract (500 mg/kg) supplementation demonstrated the strongest lipid-normalizing effects in comparison with the positive group manifested by a significant decrease (*p* < 0.01) in triglycerides, total cholesterol, and LDL (75.3 ± 3.1, 105.2 ± 3.3, and 45.1 ± 4.3) mg/dL and significant decrease (*p* < 0.05) in total lipid (376.3 ± 20.6) mg/dL. In addition, the mean value of VLDL in the BN sprout extract was close to the mean value of the control negative group.

### 3.5 Effect of BN seed and BN sprout extracts on serum liver enzymes

**Figure 2:**
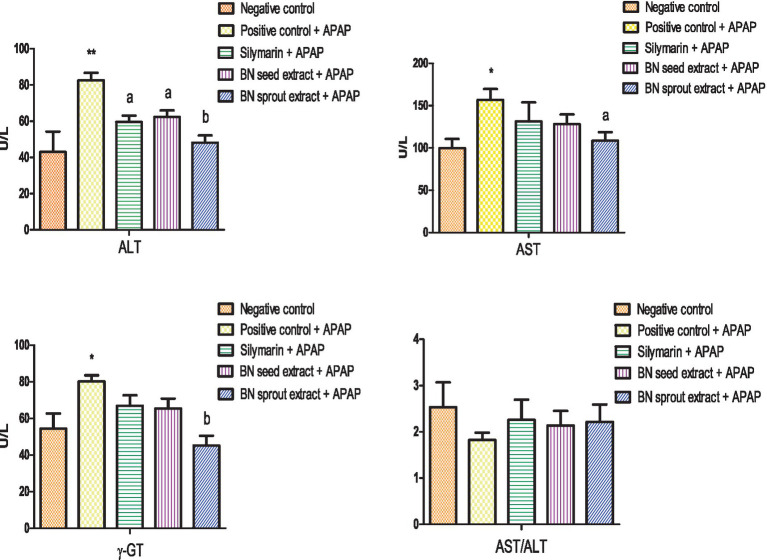
Effect of BN seed and BN sprout extracts on liver enzymes (ALT, AST, *γ*-GT levels, and AST/ALT). The bars point out the means ± SE (*n* = 10 per group). The signals (* and **) indicate significant differences at (*p* < 0.05 and *p* < 0.01, respectively) compared to the negative control group. The letters (a and b) indicate significant differences at *p* < 0.05 and *p* < 0.01, respectively, compared to the APAP-positive control group (one-way ANOVA followed by Tukey’s HSD *post-hoc* test). APAP, paracetamol; BN, *Brassica nigra*; ALT, alanine aminotransferase enzyme; AST, aspartate aminotransferase enzyme; *γ*-GT, *γ*-Glutamyl transferase.

Serum ALT, AST, and *γ*-GT levels significantly increased in the positive control group after receiving a single oral dose of APAP (82.54 ± 4.16 U/L, *p* < 0.01; 156.7 ± 12.9 U/L, *p* < 0.05; and 80.22 ± 3.31 U/L, *p* < 0.05), suggesting hepatotoxicity, in contrast to the negative group (Figure 2). Serum ALT levels were significantly lower (*p* < 0.05) in the silymarin-treated group than in the positive control. There was no statistically significant decrease in *γ*-GT or AST levels following silymarin therapy. The group supplemented with 500 mg/kg BN seed extract showed a significant reduction in ALT (62.31 ± 3.62 U/L, *p* < 0.05), with non-significant decreases in AST and *γ*-GT. Impressively, BN sprout extract at 500 mg/kg demonstrated the most pronounced hepatoprotective effect. It produced a highly significant normalization of *γ*-GT (45.18 ± 5.27 U/L, *p* < 0.01), representing a 43.7% reduction compared to the positive control group. Additionally, BN sprout extract significantly reduced ALT (48.10 ± 3.99 U/L, *p* < 0.01) and AST (108.5 ± 10.0 U/L, *p* < 0.05). The marked reduction in *γ*-GT is particularly noteworthy, as this enzyme is a sensitive indicator of cholestasis and oxidative stress-induced biliary injury. There was no change in the AST/ALT ratio across all experimental groups.

### 3.6 Effect of BN seed and BN sprout extracts on some liver metabolites, serum TP, Alb, glob, Alb/glob ratio, T-BIL, D-BIL, and BUN

The effect of BN seed and sprout extracts on serum total protein parameters is presented in Table 5. Administration of 3 g/kg APAP markedly reduced total protein (TP) levels (6.61 ± 0.51 g/dL) compared with the negative control (8.93 ± 0.62 g/dL, *p* < 0.05). The positive control group exhibited a marked reduction in Alb, Glob, and albumin/globulin (A/G) ratio, though these changes were statistically insignificant. Silymarin treatment (100 mg/kg) and BN seed extract (500 mg/kg) provided partial protection, as TP (6.85 ± 1.17 g/dL and 6.75 ± 0.95 g/dL), respectively. Alb concentrations in the two groups remained lower than those in the negative control, but they were not significantly different from those in the APAP group. However, the Glob concentrations and A/G ratio were not significantly affected in any of the treatment groups. In contrast, BN sprout extract (500 mg/kg) significantly (*p* < 0.05) restored the serum protein level, reaching a value (8.75 ± 0.51 g/dL) close to that of the negative control group (8.93 ± 0.62 g/dL. Similarly, the ALB level in the BN sprout group was significantly elevated (*p* < 0.05) compared to the positive control group (4.89 ± 0.45 g/dL vs. 3.10 ± 0.43 g/dL).

**Table 5:** Effect of BN seed and BN sprout extracts on liver metabolites.

Group	TP g/dL	Alb g/dL	Glob g/dL	Alb/Glob ratio	T-Bil umol/L	D-Bil umol/L	BUN mmol/L
Negative control	8.93 ± 0.62	4.67 ± 0.81	4.06 ± 0.66	1.19 ± 0.17	9.53 ± 1.34	5.13 ± 0.50	7.91 ± 0.47
Positive control + APAP	6.61 ± 0.49*	3.10 ± 0.43	3.37 ± 1.22	0.90 ± 0.13	16.43 ± 1.11*	7.83 ± 0.61*	7.28 ± 1.02
Silymarin + APAP	6.85 ± 1.17	3.17 ± 1.50	3.56± 0.72	0.87± 0.12	9.54 ± 2.43	4.37± 0.41^a^	7.74± 0.51
BN seed extract + APAP	6.67 ± 0.95	3.45 ± 0.91	3.31± 1.32	1.08± 0.17	9.04 ± 3.54	5.71± 0.79	6.49± 0.44
BN sprout extract + APAP	8.78 ± 0.51^a^	4.89 ± 0.45^a^	4.0 ± 0.72	1.12 ± 0.11	10.74 ± 1.90	4.16 ± 0.58^a^	7.83 ± 0.42
*p*-value	0.0482	0.0475	0.1378	0.1854	0.0453	0.0468	0.1538

Values represent the mean ± standard error (SE), (*n* = 10 per group). Within a column, values marked with a signal * differ significantly (*p* < 0.05) from the negative control, while values marked with a superscript^a^ differ significantly (*p* < 0.05) from the APAP-positive control. APAP, paracetamol; BN, *Brassica nigra*. TP, total protein; Alb, albumin; Glob, globulin. T-Bil, total bilirubin; D-Bil, direct bilirubin; BUN, blood urea nitrogen.

Administration of APAP increased significantly (*p* < 0.05) both T-Bil and D-Bil levels (16.43 ± 1.11 and 7.83 ± 0.61, respectively) compared to the negative control, indicating liver dysfunction (Table 5). Treatment with silymarin and BN sprouts extract significantly (*p* < 0.05) decreased D-Bil levels compared to the APAP group (4.37 ± 0.41 and 4.16 ± 0.58 vs. 7.83 ± 0.61, respectively). However, BN seeds extract (500 mg/kg) showed insignificant improvement in T-Bil or D-Bil levels compared to the APAP group. BUN levels did not differ among the experimental groups.

### 3.7 Effect of BN seed and BN sprout extracts on hepatic antioxidant activities and malondialdehyde

**Figure 3:**
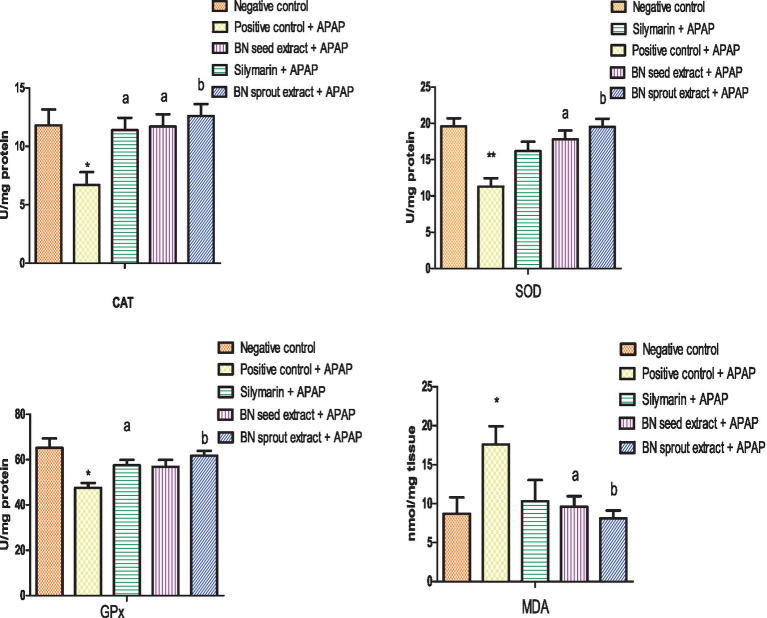
Effect of BN seed and BN sprout extracts on antioxidant activities and malondialdehyde content in liver tissue. The bars point out the means ± SE (*n* = 5 per group). The signals (* and **) indicate significant differences at (*p* < 0.05 and *p* < 0.01, respectively) compared to the negative control group. The letters (a and b) indicate significant differences at *p* < 0.05 and *p* < 0.01, respectively, compared to the APAP-positive control group (one-way ANOVA followed by Tukey’s HSD *post-hoc* test). APAP, paracetamol; BN, *Brassica nigra*; CAT, catalase; SOD, superoxide dismutase; GPx, glutathione peroxidase; MAD, malondialdehyde.

The positive control, administered with 3 g/kg APAP, showed a significant decrease in antioxidant enzymes, including CAT, GPx, and SOD (6.7 ± 1.10, 47.6 ± 2.12, *p* < 0.05, and 11.3 ± 1.15 U/mg protein, *p* < 0.01, respectively) compared to the negative control group (Figure 3). MDA, an indicator of lipid peroxidation, increased by about 50% compared to the negative control (17.6 ± 2.32 nmol/mg tissue, *p* < 0.05). The findings indicated that APAP induced oxidative stress in the liver tissue. Silymarin supplementation restored CAT and GPx levels (11.4 ± 1.06 and 57.5 ± 2.43 U/mg protein, respectively; *p* < 0.05), and partially improved SOD activity. It also decreased MDA, though these latter changes were not statistically significant. The group receiving 500 mg/kg BN seed extract showed a significant enhancement in CAT and SOD activities (11.7 ± 1.06 and 17.8 ± 1.24 U/mg protein, respectively; *p* < 0.05) but had an insignificant effect on GPx. Notably, the 500 mg/kg dose of BN sprout extract exhibited the most potent antioxidant activity, as evidenced by significantly higher activities of CAT (12.6 ± 1.03 U/mg protein), SOD (19.5 ± 1.14 U/mg protein), and GPx (61.76 ± 2.16 U/mg protein) compared to other groups (*p* < 0.01). BN sprout extract normalized MDA, highly significant at *p* < 0.01, whereas MDA normalization was at *p* < 0.05 for BN seed extract.

### 3.8 Effect of BN seed and BN sprout extracts on apoptosis (CK18 and CCCK-18)

**Figure 4:**
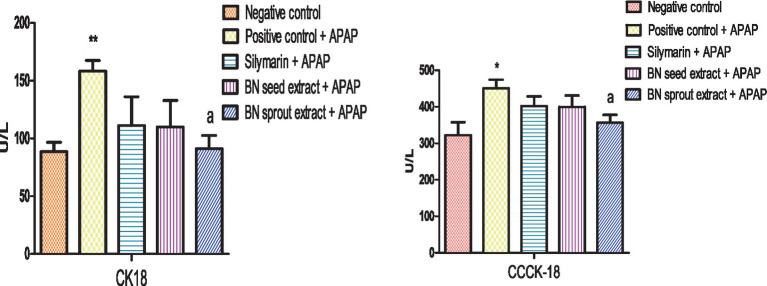
Effect of BN seed and BN sprout extracts on apoptosis (CK18 and CCCK-18) content. The bars point out the means ± SE (*n* = 5 per group). The signals (* and **) indicate significant differences at (*p* < 0.05 and *p* < 0.01, respectively) compared to the negative control group. The letter (a) indicates significant differences, *p* < 0.05, compared to the APAP-positive control group (one-way ANOVA followed by Tukey’s HSD *post-hoc* test). APAP, paracetamol; BN, *Brassica nigra*; CK18, cytokeratin-18; CCCK-18, caspase-cleaved cytokeratin.

Administration of APAP significantly elevated both CK18 (*p* < 0.01) and CCCK-18 (*p* < 0.05) in relation to the negative control (158.22 ± 9.32 and 450.67 ± 23.71 vs. 88.51 ± 8.23 and 322.43 ± 35.61), respectively, confirming hepatocellular apoptosis and necrosis (Figure 4). Treatment with silymarin or BN seed extract moderately reduced CK18 and CCCK-18 levels, in contrast to the APAP-positive group. BN sprout extract significantly decreased both CK18 and CCCK-18 levels (*p* < 0.05) relative to the APAP group (91.32 ± 11.33 and 356.38 ± 21.72 vs. 158.22 ± 9.32 and 450.67 ± 23.71), respectively, returning them toward the values observed in the negative control.

### 3.9 Histopathological examination

**Figure 5:**
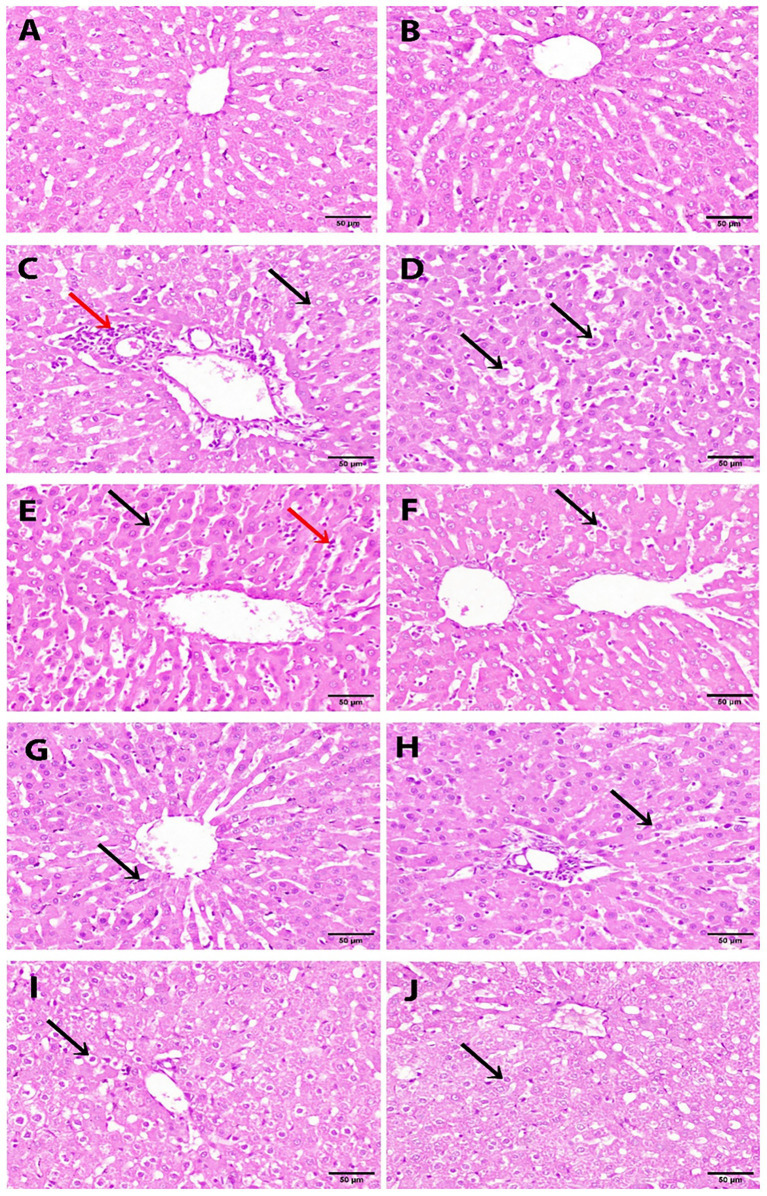
Histopathological analysis of liver tissues stained with hematoxylin and eosin (H&E). Scale bar = 50 μm (400× magnification). (*n* = 3 per group). **(A,B)** Negative control group showing normal hepatic lobule architecture. **(C,D)** Positive control (APAP-treated) group showing hepatocellular vacuolar degeneration (black arrow) and cholangitis (red arrow) in **(C)**, and hepatocellular apoptosis (black arrow) in **(D)**. **(E,F)** Silymarin-treated group showing activated Kupffer cells (black arrow) and a few leukocytes in the hepatic sinusoids (red arrow) in **(E)**, and a few leukocytes in the sinusoids (black arrow) in **(F)**. **(G,H)** The BN seed extract-treated group shows slight Kupffer cell activation (black arrow) in **(G)** and slight vacuolar degeneration in sporadic hepatocytes (black arrow) in **(H)**. **(I,J)** The BN sprout extract-treated group showed a few hepatocytes with hydropic degeneration (black arrows). APAP, paracetamol; BN, *Brassica nigra*.

Microscopic observation of liver specimens proved that the negative control group had a normal histological architecture of the hepatic lobule (Figures 5A,B). Conversely, the positive control group treated with APAP showed typical hepatotoxicity, marked by hepatocellular vacuolar degeneration, cholangitis (Figure 5C), and hepatocellular apoptosis (Figure 5D). Histological observation of the liver sample from rats treated with silymarin (drug reference) showed Kupffer cell activation and the presence of a few leucocytes in the hepatic sinusoids (Figures 5E,F). Liver tissue from rats administered BN seed extract demonstrated slight Kupffer cell activation (Figure 5G) and slight vacuolar degeneration in sporadic hepatocytes (Figure 5H). Meanwhile, liver tissue from rats supplemented with BN sprout extract showed few hydropic degenerations in some hepatocytes (Figures 5I,J).

## 4 Discussion

The marked increase in both absolute and relative liver weight recorded in the group administered by APAP confirms hepatotoxicity induction as reported previously (49), who stated that overdose of paracetamol is the main cause of acute liver failure. Increasing liver weight (absolute and relative) owing to APAP, which induces mitochondrial swelling, contributes to hepatocyte enlargement and increased liver weight in early APAP toxicity (10). In rats, a dose of more than 500 mg/kg APAP causes hepatocyte swelling and inflammation within 24 to 48 h. This initial phase increases liver weight due to inflammation and vascular leakage, resulting in blood pooling (50). The significant reduction in liver weight observed in rats pretreated with BN seed and BN sprout extracts may be attributed to multiple protective mechanisms. First, the antioxidant properties of BN extracts, particularly the bioactive compounds identified by HPLC (catechin, ferulic acid, rutin, and quercetin), likely mitigated APAP-induced oxidative stress, thereby preserving mitochondrial integrity and preventing hepatocyte swelling (51, 52). Second, the anti-inflammatory effects of these phytochemicals may have attenuated inflammatory cell infiltration and vascular leakage, both of which contribute to liver enlargement (53). The observed reduction in relative liver weight recorded with BN seed and BN sprout extracts agrees with the findings of Salman et al. (39), who demonstrated that a 14-day pretreatment with BN seed extract significantly decreased relative liver weight. On the other hand, differences between the two extracts for these parameters were not statistically significant; BN sprout extract numerically achieved the lowest absolute and relative liver weights, consistent with recent findings that sprout extracts exert enhanced protective effects (54).

The present results indicated that APAP-induced liver injury impairs liver function, disrupts lipid metabolism, and alters lipid profile, as shown by elevation of triglyceride, total cholesterol, LDL-C, total lipid, and VLDL, agrees with a recent study (55), which concluded that oral supplementation of APAP caused an substantial changes in lipid profile, evidenced by an sharp rise in TG, TC, LDL, VLDL, and TL. The finding proved that APAP induced hepatoxicity, as shown in a recent study (56), which reported that acute liver injury causes an abnormal serum lipid profile. This dyslipidemia resulting from APAP may be attributed to its conversion to the toxic metabolite NAPQI, which depletes glutathione, binds to liver proteins, and increases oxidative stress (57). Administration of 500 mg/kg BN seed extract produced the same effect as silymarin on serum lipid profiles; both treatments decreased total cholesterol and LDL, while silymarin also reduced triglycerides, consistent with previous research findings (58). Unfortunately, little has been reported on the impact of BN seeds and sprouts on the lipid profile; therefore, evidence from the broader Brassicaceae family will be cited. Several studies have demonstrated that cruciferous sprouts reduce hepatic cholesterol levels and improve oxidative state (4, 59). *Brassica juncea* leaf extract decreased serum levels of triglyceride, TC, and LDL significantly (60). The strongest lipid-normalizing effects were achieved with 500 mg/kg BN sprout extract, which significantly reduced triglycerides, total cholesterol (TC), LDL, and total lipids (TL); notably, TC and LDL levels returned nearly to normal, consistent with previous findings (59). The clear effect of sprouts might be attributed to sulforaphane SFN, which is abundant in cruciferous vegetables (61). SFN has several health benefits, including antioxidants and anti-inflammatory properties (19). Plasma SFN concentration was 7-fold higher in subjects who received fresh broccoli sprouts than in those who received broccoli supplements (6). Additionally, cruciferous sprouts have been shown to adjust cholesterol metabolism and reduce oxidative stress (59).

To assess hepatotoxicity, the following enzymatic biomarkers were selected as indicators in this study: ALT, AST, and *γ*-GT (62). The notable increase in serum levels of ALT, AST, and *γ*-GT observed in the APAP-treated group confirms previous research findings (63), which reported elevated serum enzymes, including ALT, AST, *γ*-GT, and LDH, following paracetamol treatment. Between 24 and 72 h after APAP intake, liver enzyme levels can elevate to several thousand (64). APAP overdose-induced hepatocyte degeneration and hepatic injury enzymes (65) contributed to reactive oxygen species (ROS), DNA damage, and oxidative stress (66). APAP compromises the structural integrity of the liver, particularly by increasing membrane permeability, leading to the leakage of enzymes into the bloodstream, which is an indicator of hepatotoxicity (3). According to Chidiac et al. (67), overdose of APAP exhausted glutathione storage, accumulated NAPQI, leading to mitochondrial dysfunction and oxidative damage.

The substantial drop in serum ALT and the moderate decreases in AST and *γ*-GT observed in the group receiving 500 mg/kg BN seed extract align with recent findings (39). Several studies have indicated that BN treatment restores liver enzyme levels compared to control groups (18, 24). Pretreatment for 3 weeks with BN seed aqueous extract significantly decreased the serum levels of SGOT, SGPT, and *γ*-GT (43). *Brassica juncea* extract served as a prophylactic and/or treatment, significantly reducing the elevation of transaminases (68). On the other hand, the present study confirmed that a 500 mg/kg dose of BN sprouts extract improved serum liver enzymes more effectively than BN seed extract. This was evidenced by a pronounced decline in ALT and *γ*-GT, with a notable decrease in AST. As no studies have directly compared BN seeds with BN sprouts, and research on BN sprouts remains limited, the present discussion draws on findings from other members of the Brassicaceae family. The present finding on BN sprout extract is consistent with a recent study Shahin et al. (59), which reported that daily consumption of broccoli seeds and sprouts reduces ALT and ALP levels, supporting their potential role in preventing hepatic damage. Serum ALT, *γ*-GT, and ALP levels significantly decreased in patients after daily intake of broccoli sprout extract (69). Pretreatment with allyl isothiocyanate (AITC), a bioactive molecule found in cruciferous plants, exerts a protective effect against APAP-induced hepatotoxicity by reducing serum AST and ALT levels (9). The pronouncing effect achieved by BN sprouts as a protective agent in the injured liver, owing to sprouting, can enhance the concentration and bioavailability of bioactive phytochemicals in Brassica sprouts (5, 27), along with the synthesis of new phenolic compounds (22). Previous studies have demonstrated that *Brassica nigra* sprouts contain significant levels of glucoraphanin, the precursor to SFN (70, 71). Upon hydrolysis, glucoraphanin is converted to SFN, which is recognized as one of the most potent inducers of the Nrf2 pathway among isothiocyanates (6, 9). The enrichment of glucosinolates during sprouting has been well documented in various *Brassica* species, suggesting that the enhanced bioactivity of BN sprouts may be partially mediated through SFN-dependent Nrf2 activation (5, 27).

Total protein, albumin, and globulin levels reflect liver function, as protein metabolism and synthesis are key to hepatic processes. Higher levels of direct bilirubin in the blood indicate hepatic cell damage or disease (72). The reduction in serum TP levels observed in the APAP-treated group in the existing study is consistent with previous studies (73). Post administration of an overdose of paracetamol, there were reductions in serum TP, Alb, and uric acid (74). APAP administration induces acute hepatocellular damage and reduces hepatocyte number, which may consequently impair the liver’s protein-synthesizing capacity (41, 75). The marked reduction in serum Alb in the APAP group may be attributed to increased vascular permeability, which allows albumin to leak into the intercellular spaces (76). The present results indicate that BN sprout extract exerts a more pronounced hepatoprotective effect than BN seed extract, as it restored the serum protein and Alb levels. These findings are consistent with a previous study (4), which reported that sprout consumption can increase overall serum protein levels, thereby enhancing liver detoxification. A 2% *Brassica oleracea* extract diet significantly increased TP, Alb, and the Alb/Glob ratio, while globulin levels remained unchanged (77). Pretreatment with BN extract for 3 weeks increased serum albumin and the TP levels compared with the intoxicated rats; meanwhile, globulin levels did not change significantly (75). The significant increase in T-Bil and D-Bil observed in the overdose APAP positive group aligns with recent work (55, 78), which states that APAP-induced toxicity causes serum hyperbilirubinemia due to excessive heme destruction and blockage of the biliary tract. In APAP-treated rats, hepatotoxicity was associated with significant elevations in serum T-Bil and D-Bil (72, 75). The significant reduction in D-Bil levels in the groups supplemented with silymarin and BN sprouts extract is consistent with previous findings (69), which reported that intake of sulforaphane-rich broccoli sprout extract improves serum albumin and bilirubin levels. However, the slight decrease in T-Bil and D-Bil levels in the BN seed extract group aligns with previous studies (18, 43). *Brassica juncea* extract, as a preventive or therapeutic measure, resulted in a decrease of T-Bil compared to hepatic intoxication (68).

ROS and free radicals are strongly linked to various degenerative diseases (79). The rise in reactive oxygen species (ROS) may result from either overproduction or antioxidant enzyme activity reduction (80). The exciting study depends upon CAT, SOD, and GPx as indicators of hepatic cellular redox homeostasis. The oxidative stress induced by an APAP overdose, evidenced by a significant decrease in antioxidant enzyme activities and a marked elevation of MDA in hepatic tissue, is consistent with recent research (65, 78). APAP-induced hepatotoxicity is attributed to the conversion of APAP to NAPQI, which depletes glutathione, thereby amplifying oxidative stress, mitochondrial dysfunction, DNA damage, and disruptions in cellular redox equilibrium (57, 81). Pretreatment with BN (seed and sprout) extracts reduced oxidative stress caused by APAP administration, showing varying degrees of improvement. However, BN sprout extract demonstrated greater effectiveness in restoring hepatic CAT, SOD, and GPx, accompanied by a decrease in MDA, which agrees with recent work by Aljutaily et al. (54), even though their study focused on the nephroprotective effects of BN sprouts. Unfortunately, few studies have been conducted on BN sprouts; therefore, the present research was forced to cite studies on sprouts from the Brassica family. Broccoli sprouts have strong antioxidant effects, containing 10–100 times more enzymes than ungerminated vegetables (82). The improvement in redox status observed in the present study following BN extract treatment may be attributed to its phytochemically active compounds, such as SFN. Although SFN was not directly measured in our study, previous investigations have demonstrated that SFN may mitigate oxidative stress and glutathione depletion by activating the Nrf2 pathway, thereby enhancing antioxidant activity (83). This suggests that the significant enhancement of antioxidant activities (CAT, SOD, and GPx) observed with BN sprout extract may be mediated, in part, through Nrf2 activation. The superior restoration of all three antioxidant enzymes by BN sprouts, particularly GPx, which was not significantly improved by seeds, suggests more robust Nrf2 activation by the sprout extract. Moreover, dramatic increases in bioactive flavonoids such as quercetin and rutin may also contribute to Nrf2 activation (53, 84).

Caspase-cleaved cytokeratin-18 (CCCK-18) is a fragment of the protein cytokeratin 18 (CK18) that is released during apoptosis, a form of programmed cell death (85). CK18 and CCCK-18 are sensitive biomarkers of apoptotic cell death with potential for early detection of apoptosis in drug-induced liver injury, including paracetamol-induced hepatotoxicity (86). A significant elevation in CK18 (*p* < 0.01) and CCCK-18 (*p* < 0.05) was observed in the APAP-treated positive control group compared to the negative control group. This observation aligns with a recent study by Scullion et al. (87), which reported a significant increase in CK18-Asp396 (a fragment of CK-18) and CK18 after 4 h of paracetamol ingestion, indicating early liver damage. These findings confirm that hepatocyte apoptosis is associated with APAP overdose, as previously reported (88), and that CK18 and CCCK-18 are released during this process. To date, the precise impact of Brassica plants on serum CK18 and CCCK-18 levels following liver injury has not been directly studied. Notably, the reduction in these markers observed with Brassica treatment in the present study indicates a hepatoprotective effect. Treatment with silymarin or BN seed extract moderately lowered CK18 and CCCK-18 levels, suggesting these two extracts provide partial hepatoprotection. In contrast, BN sprout extract significantly restored both CK18 and CCCK-18 levels, bringing them close to normal values. This suggests that BN sprouts provide superior anti-apoptotic effects against APAP-induced hepatocyte injury compared with BN seed extract, consistent with previous studies demonstrating that sprouting enhances functional activities such as antioxidation and anti-inflammation (89).

In the present study, this enhanced functionality was evident across multiple parameters: BN sprouts produced significantly greater improvements in antioxidant enzyme activities (CAT, SOD, and GPx), liver enzyme profiles (ALT, AST, and *γ*-GT), and apoptotic markers (CK18 and CCCK-18) compared to BN seeds. These findings align with the broader literature indicating that sprouting enriches the concentration and bioavailability of bioactive phytochemicals, thereby potentiating a range of beneficial biological activities (5, 22, 26). Notably, the significant reduction in CK18 and CCCK-18 levels observed with BN sprout extract indicates inhibition of the caspase cascade that executes apoptotic cell death. Apoptosis is regulated by the balance between pro-apoptotic (Bax, Bak) and anti-apoptotic (Bcl-2 and Bcl-xL) proteins, which control mitochondrial outer membrane permeabilization (MOMP) and subsequent cytochrome c release (10). Cytochrome c activates caspase-9, which in turn activates executioner caspase-3, leading to the cleavage of cytokeratin-18 (CCCK-18) and other cellular substrates.

Histological examination of liver specimens confirmed the findings of this study. Animals in the negative control group exhibited normal liver architecture; meanwhile, rats in the APAP-treated positive control group showed severe hepatotoxicity, characterized by hepatocellular vacuolar degeneration, cholangitis, and apoptosis. These observations were supported by all measured parameters, confirming that APAP induces hepatotoxicity, as reported in recent studies (55, 57, 67). Rats in the groups treated with silymarin and BN seed extract exhibited a moderate hepatoprotection, as evidenced by slight Kupffer cell activation and mild vacuolar degeneration in sporadic hepatocytes. Supplementation with BN sprout extract conferred superior hepatoprotection against APAP-induced hepatotoxicity, as evidenced by minimal histopathological changes in hepatic tissue, consistent with previous studies on sprout supplementation (4, 59, 71).

Collectively, the present results cannot be ignored in terms of the hepatoprotective effect of BN seed and BN sprout extracts against APAP-induced liver injury. Nevertheless, insight from the overall findings clearly indicated that BN sprout extract possesses superior hepatoprotective capacity compared to BN seed extract. The pronounced benefits of BN sprouts extract as a natural hepatoprotective agent may be attributed to the enrichment of total phenols, antioxidant activity, and phytochemicals during the sprouting process, as recognized in this study, resulting from increased enzyme levels that liberate the conjugated phenolic compounds and synthesize new ones (22). Sprouting may increase the concentration and bioavailability of antioxidant compounds such as sulforaphane, sulforaphene, ITCs, and GSLs in Brassica sprouts (5, 27), along with other nutrients, such as vitamins, minerals, and essential amino acids (4). Sprout also breaks down complex macromolecules into simpler, more digestible forms (25). Additionally, BN sprouts extract stronger antioxidants and anti-apoptotic effects. The superior efficacy of BN sprout extract compared to its seed counterpart, as demonstrated by greater normalization of liver enzymes, it uniquely demonstrated significant reductions in *γ*-GT, whereas, BN seeds showed no significant effect on this parameter, restoration of all three major antioxidant enzymes (CAT, SOD, and GPx), whereas, BN seeds significantly improved only CAT and SOD, and modulation of apoptosis, as BN sprouts significantly reduced both CK18 and CCCK-18 levels, bringing them close to normal values, whereas, BN seeds had non-significant effect. Moreover, the phytochemical compounds detected by HPLC in BN seeds and BN sprouts in this study revealed that an abundance of bioactive constituents, particularly catechin, ferulic acid, rutin, and quercetin, supports the hepatoprotective effects documented in BN sprouts rather than BN seeds. Notably, gallic acid was detected exclusively in BN sprouts, while absent from BN seeds. Gallic acid possesses well-documented antioxidants, anti-inflammatory, and anti-apoptotic properties (90). The exclusive presence of gallic acid in sprouts may contribute to the superior restoration of GPx and the significant reduction in apoptosis markers observed with BN sprouts (91). Quercetin constitutes a more than 30-fold increase in BN sprout than that present in BN seeds, which could lead to superior restoration of GPx, the only antioxidant enzyme not significantly improved by seeds (84). They concluded that quercetin has been shown to upregulate GPx expression through Nrf2-dependent mechanisms. Quercetin modulates cell death mechanisms and reduces liver fibrosis, highlighting its therapeutic benefits (84). In a mouse model of metabolic dysfunction-associated fatty liver disease, quercetin exhibits hepatoprotective effects by regulating hepatic lipid metabolism, maintaining redox balance, and suppressing inflammatory responses (92). Catechin’s potent anti-inflammatory, antioxidant, and antiangiogenic qualities may shield the liver from acetaminophen-induced damage (52). The hepatoprotective action of catechin might involve radical scavenging and cytokine suppression (93). Ferulic acid successfully stops oxidative damage to liver tissue in non-alcoholic liver disease (51). The superior efficacy of BN sprout extract compared to its seed counterpart, as demonstrated by its superior lipid-normalizing effects, particularly the significant reduction in triglycerides, which was not achieved by the seed extract, may be partially attributed to its higher ferulic acid content (94). Additionally, ferulic acid has been reported to reduce *γ*-GT levels by mitigating oxidative stress in the biliary epithelium (95), consistent with the unique results obtained in the present study, in which BN sprouts normalized *γ*-GT. Rutin is considered a potential and effective supplementary therapy to prevent hepatotoxicity and liver injury caused by prolonged or daily use of VORTX (96). Furthermore, BN sprouts extract restored all three major antioxidant enzymes (CAT, SOD, and GPx), whereas BN seed extract improved only CAT and SOD, owing to the high antioxidant capacity detected in this work. The anti-apoptotic and antioxidant effects support the therapeutic benefits of rutin. Rutin has been shown to inhibit caspase-3 activation and reduce CK18 cleavage in models of drug-induced liver injury (53, 96). The significant reduction in CCCK-18 levels observed only with BN sprouts may be partially attributed to the higher rutin content (53).

An additional consideration supporting the investigation of BN sprouts is the anti-nutritional factors present in raw BN seeds. Seeds of the Brassicaceae family contain compounds such as phytic acid, tannins, and high levels of glucosinolates, which, when hydrolyzed, may produce goitrogenic and cytotoxic effects at elevated concentrations (28). Sprouting has been shown to significantly reduce these anti-nutritional factors through enzymatic degradation and leaching during the germination and rinsing processes (25). This suggests that sprouting not only enhances bioactive phytochemicals but also mitigates the potential adverse effects associated with raw seed consumption. These findings support the potential of BN sprouts as a safer and more effective hepatoprotective agent, though future toxicological studies are warranted to confirm long-term safety.

The present study makes several novel contributions to the literature. First, it provides the first direct comparative assessment of the hepatoprotective efficacy of *Brassica nigra* seeds and sprouts, demonstrating that the sprout form exhibits superior activity across multiple parameters. Second, it establishes, for the first time, the hepatoprotective potential of BN sprouts, a previously unexplored form of this plant. Third, it presents the first comprehensive comparative HPLC profiling of phenolic and flavonoid compounds in BN seeds versus sprouts, revealing qualitative differences (e.g., gallic acid detected only in sprouts) that may underpin the enhanced bioactivity of sprouts. Fourth, it demonstrates that BN sprouts exert significant anti-apoptotic effects, as evidenced by reduced CK18 and CCCK-18 levels, a mechanism not previously reported for BN.

## 5 Conclusion

The existing study has demonstrated that a single overdose of APAP induces acute hepatotoxicity in Wistar rats. The results provide the first evidence that BN sprout extract possesses superior and unique advantages over BN seed extract in protecting the liver against APAP-induced hepatotoxicity, compared to BN seed extract. The significant hepatoprotective benefit of the sprout extract is evidenced by several key findings, including the normalization of the lipid profile, particularly significant reductions in triglyceride, total cholesterol, low-density lipoprotein, and total lipid levels, restoration of hepatic antioxidant enzyme activities (CAT, SOD, and GPx), and reduction in lipid peroxidation (MDA). Furthermore, BN sprouts extract supplement resulted in marked improvements in serum liver enzymes, with highly significant reductions in ALT and *γ*-GT and a marked reduction in AST, as well as significant attenuation of apoptosis, as reflected by decreased levels of cytokeratin-18 (CK18) and its caspase-cleaved fragment (CCCK-18). These functional advantages are supported by qualitative and quantitative differences in phytochemical composition, including the exclusive presence of gallic acid in sprouts and higher concentrations of catechin, quercetin, ferulic, and rutin.

The present study provides a phytochemical basis for the superior hepatoprotective effects of BN sprouts and underscores the value of sprouting as a bioprocessing strategy to enhance the therapeutic potential of plant-based products. BN sprout extract confers hepatoprotection via antioxidative and anti-apoptotic pathways, likely due to increased concentrations and bioavailability of bioactive compounds following sprouting. Further clinical investigation into the safety, bioavailability, and therapeutic potential of *Brassica nigra* sprout extract is strongly merited. Future research should focus on scientific areas, particularly establishing the optimal dosing regimen; conducting pharmacokinetic studies to assess the bioavailability of key bioactive compounds such as rutin, catechin, and isothiocyanates; and elucidating upstream molecular mechanisms, including Nrf2 nuclear translocation and its transcriptional activity, assessing the expression of pro-apoptotic (Bax and Bak) and anti-apoptotic (Bcl-2 and Bcl-xL) proteins at the gene and protein levels, quantifying caspase-3 activation as a direct measure of the apoptotic cascade, and employing specific inhibitors. Such studies would provide definitive mechanistic validation of the pathways proposed in this investigation.

## Data Availability

The original contributions presented in the study are included in the article/supplementary material, further inquiries can be directed to the corresponding author.
